# Lithium-based perovskites materials for photovoltaic solar cell and protective rays window applications: a first-principle calculations

**DOI:** 10.1186/s11671-023-03790-z

**Published:** 2023-02-16

**Authors:** Muhammad Khuram Shahzad, Syed Taqveem Mujtaba, Shoukat Hussain, Muhammad Umair Farooq, Rashid Ali Laghari, Sajjad Ahmad Khan, Muhammad Bilal Tahir, Jalil Ur Rehman, Adnan Khalil, Muhammad Mahmood Ali

**Affiliations:** 1 Institute of Physics, Khwaja Fareed University of Engineering and Information Technology, Rahim Yar Khan, 64200 Pakistan; 2 Center of Theoretical and Computational Research, Khwaja Fareed University of Engineering and Information Technology, Rahim Yar Khan, Pakistan; 3 Department of Physics, Riphah International University, Faisalabad Campus, Faisalabad, Pakistan; 4 Institute of Physics, The Islamia University of Bahawalpur, Bahawalpur, 63100 Pakistan; 5 Interdisciplinary Research Center for Intelligent Manufacturing and Robotics, King Fahd University of Petroleum and Minerals, Dhahran, 31261 Saudi Arabia; 6 Centre for Mathematical Modeling and Intelligent Systems for Health and Environment (MISHE), Atlantic Technological University Sligo, Ash Lane, Sligo, F91 YW50 Ireland; 7 Department of Mechatronic Engineering, Atlantic Technological University Sligo, Ash Lane, Sligo, F91 YW50 Ireland

**Keywords:** Lithium oxide perovskite, Electronic properties, Elastic constants, Photovoltaics solar cell

## Abstract

Perovskites are the key enabler materials for the solar cell applications in the achievement of high performance and low production costs. In this article, the structural, mechanical, electronic, and optical properties of rubidium-based cubic nature perovskite LiHfO_3_ and LiZnO_3_ are investigated. These properties are investigated using density-functional theory with the aid of CASTEP software by introducing ultrasoft pseudo-potential plane-wave (USPPPW) and GG-approximation-PB-Ernzerhof exchange–correlation functionals. It is investigated that the proposed compounds exhibit stable cubic phase and meet the criteria of mechanical stability by the estimated elastic properties. Also, according to Pugh's criterion, it is noted that LiHfO_3_ is ductile and LiZnO_3_ is brittle. Furthermore, the electronic band structure investigation of LiHfO_3_ and LiZnO_3_ shows that they have indirect bandgap (BG). Moreover, the BG analysis of the proposed materials shows that these are easily accessible. Also, the results for partial density of states (DOS) and total DOS confirm the degree of a localized electron in the distinct band. In addition, the optical transitions in the compounds are examined by fitting the damping ratio for the notional dielectric functions scaling to the appropriate peaks. At absolute zero temperature, the materials are observed as semiconductors. Therefore, it is evident from the analysis that the proposed compounds are excellent candidates for solar cells and protective rays applications.

## Introduction

The simple processing method and impressive photon-to-electron system conversion efficiency of energy, perovskite solar cells materials have attracted a lot of attention as a promising 3rd-generation solar cell. It has the potential to replace traditional clean energy strategies based on solar energy conversion [1, 2]. The combination of incredible benefits, such as superb tolerance for perovskite structure defects [3], as well as the availability of outstanding efficient carriers mobility, light (photon) absorption efficiency, and sufficient carrier diffusion lifetimes, made this a strong contender for exceptional solar cell photovoltaic applications and performance [4, 5]. Since 2009, when the organic metal halide perovskite was first incorporated into the titanium dioxide (TiO_2_) structure with a moderate efficiency of 3.8 percent, perovskite materials device efficiency has 24.2 percent or surpassed this percentage, with an overall average of 24 percent certified by the Fraunhofer Institute for solar energy system [6]. Moreover, novel methodologies and technologies were used to obtain these exceptional results [7, 8]. Long-term stability has been studied in perovskite solar systems, and it was an intractable difficulty due to the lack of a corresponding standard process for perovskite materials-based photovoltaic manufacture[9–12]. The dangers of UV radiation are widely understood, and public education about the use of sunglasses, sunscreen, clothing, protective, and helmets, as well as the promotion of seeking shade and avoiding the sun during peak exposure periods, is continuing [13, 14]. Ultraviolet (UV) light is filtered out by conventional glass, but visible light, and infrared rays are still transmitted. Additional infrared radiation and UV filters can now be added thanks to recent advancements in the glass sector. The majority of these glasses are unnoticeable to the naked eye, yet they give varying degrees of infrared and UV protection [15]. Hence, a limited number of research work has been conducted yet. The designed present research work has applications in the field of photovoltaic solar cell as well as photoprotective properties of car glasses, window glasses, and sunglasses. Because it absorbs UV light, this attribute makes the proposed ceramics appealing for the manufacture of future generation electronics, such as powerless UV detectors and guards. Wind shields are made of tempered glass, which can block a significant quantity of UV; most rear windows and side are tinted but not laminate and block radiations to reach the vehicle's passengers [16–18].

Herein, the structural, electrical, optical, and mechanical properties of LiHfO_3_ and LiZnO_3_ are investigated. The GGA-PBE technique, which is embedded within the CASTEP software and designed, using DFT, is used to do complete energy estimates. Investigated compound is suitable for the photocatalytic applications like solar cell. Energy production and controlling is hot issue in these days. Many diseases are erupted due to radiations. It is necessary to protect from it. Many perovskite materials [19–21] are investigated for side window of car or room. Our investigated compound LiHfO_3_ is suitable for solar cell application because of high conductivity, low absorption and low loss function criteria. LiZnO_3_ is brittle and behaves as insulator so it totally reflects light because of low absorption capability. These compounds are novel compounds and will provide protection from radiations that can damage your body or eyes and also decrease the temperature of the room because of stoppage of radiations.

## Computational detailed

For the compound LiHfO_3_ and LiZnO_3_, the cubic structure of oxide-perovskites is considered. Compounds belong to the 221 space group. In compound, Rb atomic positions are (0.0, 0.0, 0.0) whereas Zn and Hf atomic positions at (0.5, 0.5, 0.5). In LiHfO_3_ and LiZnO_3_, the atomic positions of O are (0.0, 0.5, 0.5). Li: 1*s*^2^ 2*s*^1^, O: 2*s*^2^ 2*p*^4^, Zn: 3*d*^10^ 4*s*^2^, and Hf: 5*d*^2^ 6*s*^2^ and are the elemental configurations for the atoms in question. The CASTEP algorithm based on DFT [22] was used to determine structural, electrical, and optical characteristics. This method allows for quick computational calculations. We optimized the geometry and then calculated all of the relevant attributes. In this situation, the amount of energy per atom is 1.0 × 10^–5^eV. The remaining forces operating on atoms following geometry optimization are 0.03 eV/Å. Monkhorst–Pack grid (MPK), the *k*-integration was finished at 8 × 8 × 8 *k*-points mesh, and the cutoff energy was set at 340.0 eV over the whole Brillouin zone (BZ). For determining elastic constant, the series of phases for every strain was fixed to four and the max stress amplitudes were fixed to 0.05GPa and the maximum displacement is 0.001 Å. For geometry optimization, pressure in Giga-Pascal (GPa) is used to perceive it.

## Results and discussions

### Structural analysis

Furthermore, the structural geometries of the intended unit cell for substances are optimized. The Murnaghan state equation (MSE) is utilized to derive lattice constants while maintaining energy of crystal minimal [23]. The total quantity of energies have been computed around the volume of the balance cell as a proportion of the volume of the unit cell. The LiHfO_3_ and LiZnO_3_ have a band gap of 3.91 eV and 4.59 eV. Table 1 shows the lattice constants, volume, and bandgap of compounds LiHfO_3_ and LiZnO_3_.

**Table 1:** Compounds LiHfO_3_ and LiZnO_3_ lattice constants, volume, and bandgap

Compounds	Lattice constant (**Å**)	Volume (**Å**)^3^	Bandgap (eV)
LiHfO_3_	4.28	78.40	3.91
LiZnO_3_	3.55	44.73	4.59

**Fig. 1:**
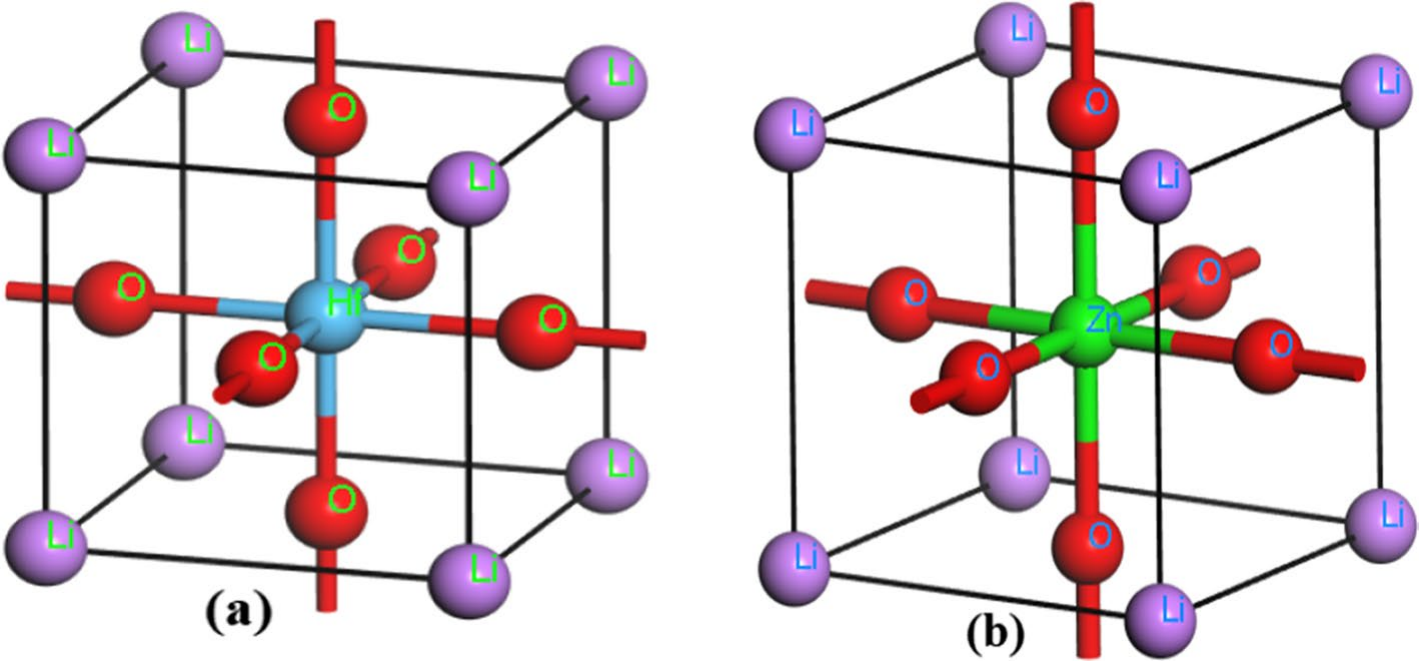
Crystal Structure of **a** LiHfO_3_ **b** LiZnO_3_ Compounds

Through geometry optimization, the optimal lattice parameters of LiHfO_3_ and LiZnO_3_ compounds are exposed to be 3.55 Å and 4.28 Å, respectively. This result is consistent with the previous results [24, 25]. This demonstrates that our first-principles computation is correct and legitimate. We also noticed there is no results data in the literature for the LiHfO_3_ and LiZnO_3_ molecule to compare. As a result, subsequent measurements will validate our measured results (Fig. 1).

### Electronic band structure and DOS

**Fig. 2:**
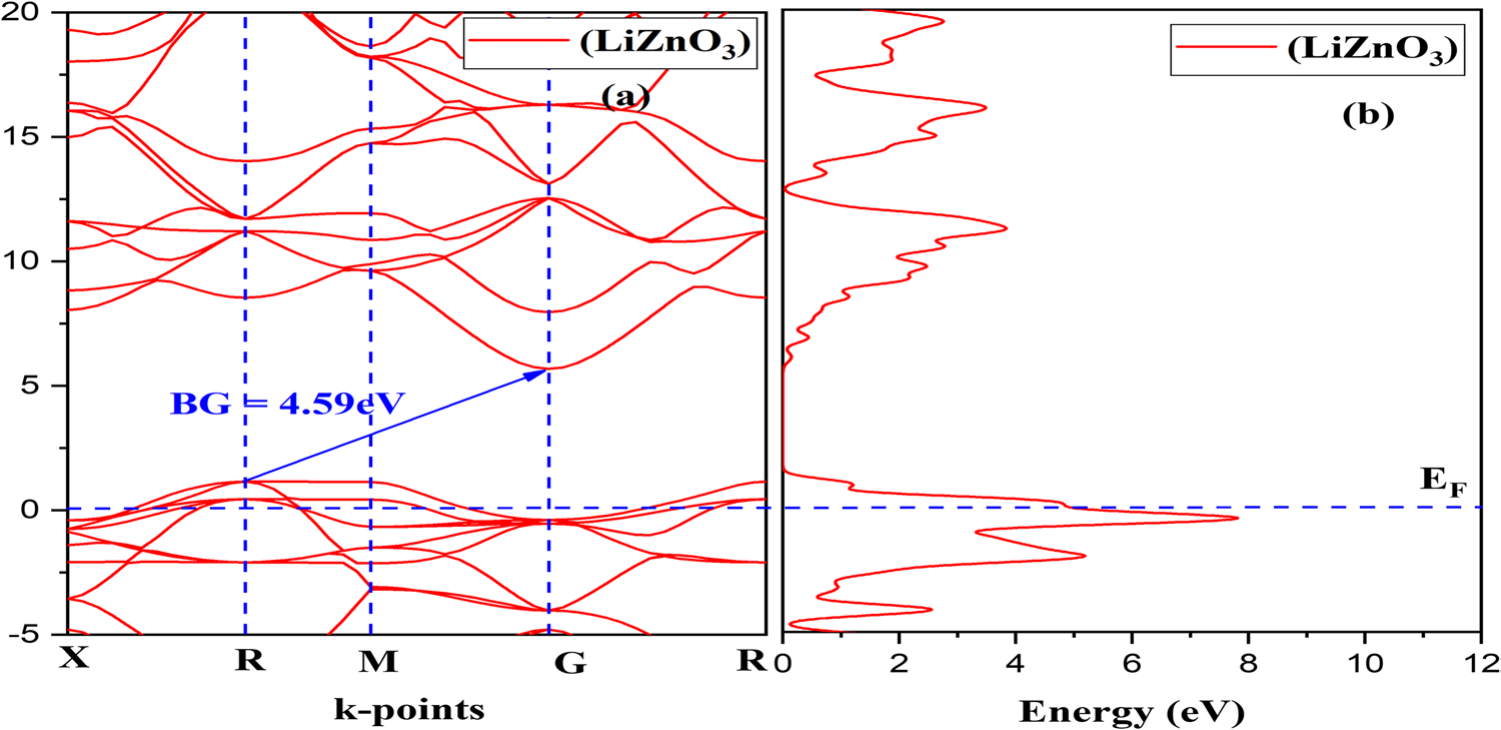
Compound LiZnO_3_ **a** Band Structure and **b** DOS

The electronic band structure offers information on the energy values in which electrons can exist and the area where no electrons are accessible. The valance band (VB) and conduction band (CB) are 2-different type of energy band. The VB lies below the free compound Fermi energy point (E_F_), while the CB is above it. The peak of the VB is designated E_F_ without considering into consideration the effect of finite-temperature because all observations were done at 0 K. The gap between the valance band minimum (VBM) and conduction band maximum (CBM) is used to compute the BG. If the VBM happens exactly upon CBM, the BG will be a direct band gap. In another instance, when the VBM and CBM are not perfectly aligned, an indirect band gap emerges. Figure 2 depicts the electronic band structure of LiHfO_3_ and LiZnO_3_. LiHfO_3_ and LiZnO_3_'s VBM and CBM are not perfectly aligned, indicating that the triple compound possesses an indirect band gap. The indirect BG value which is not exactly on top of each other is 4.59 eV and 3.91 eV of LiZnO_3_ and LiHfO_3_.

**Fig. 3:**
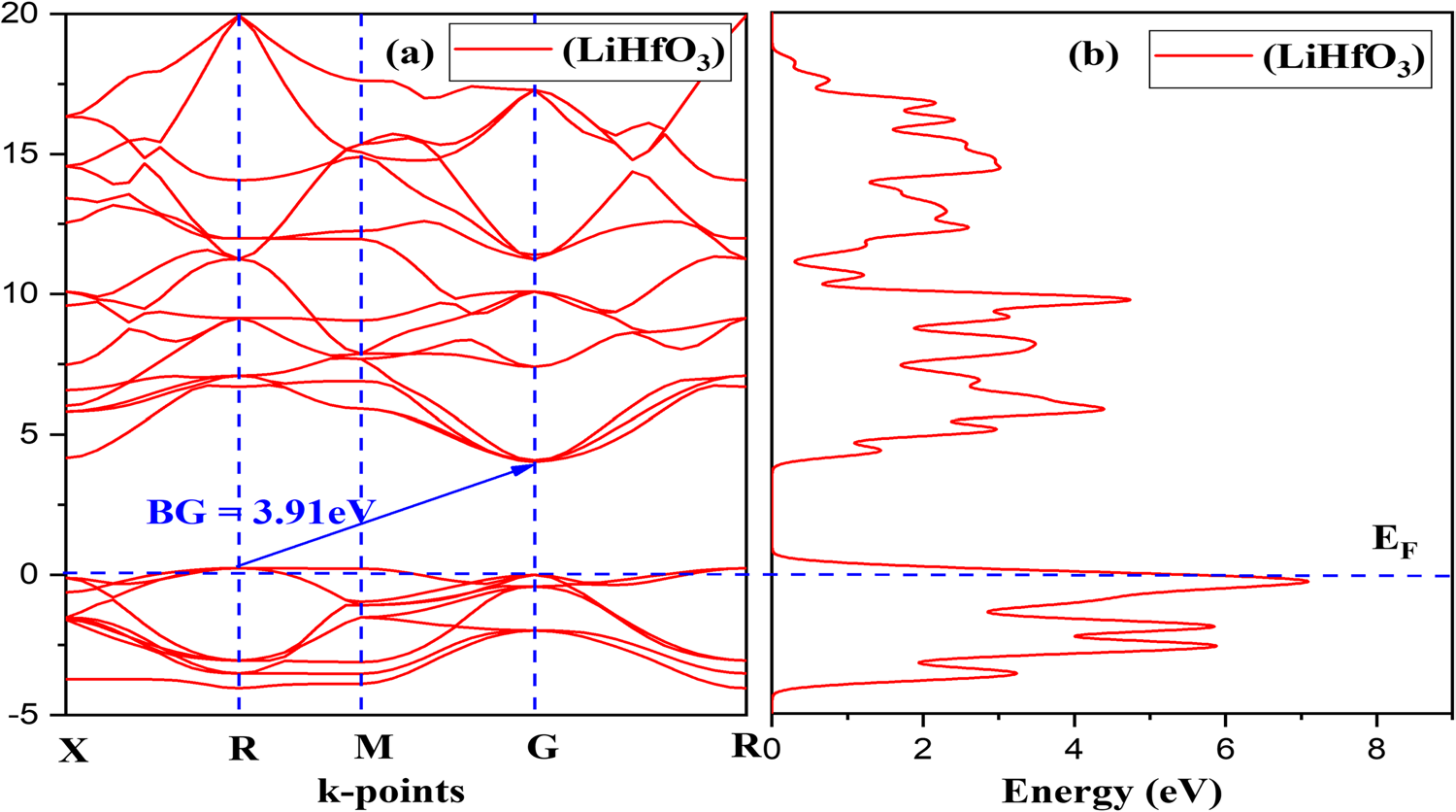
Compound LiHfO_3_ **a** Band Structure and **b** DOS

**Fig. 4:**
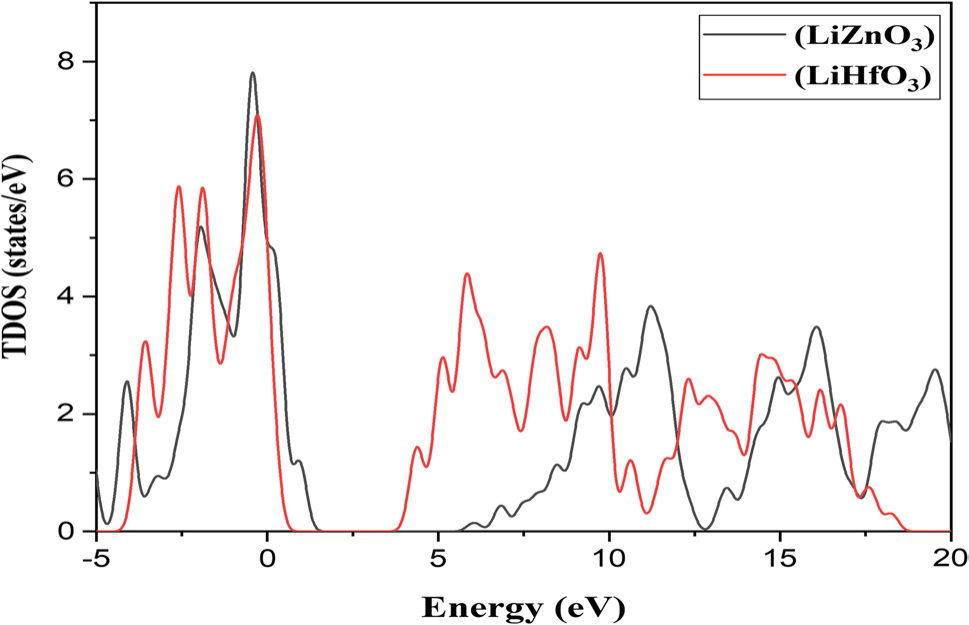
Total DOS of LiZnO_3_ and LiHfO_3_ compound

**Fig. 5:**
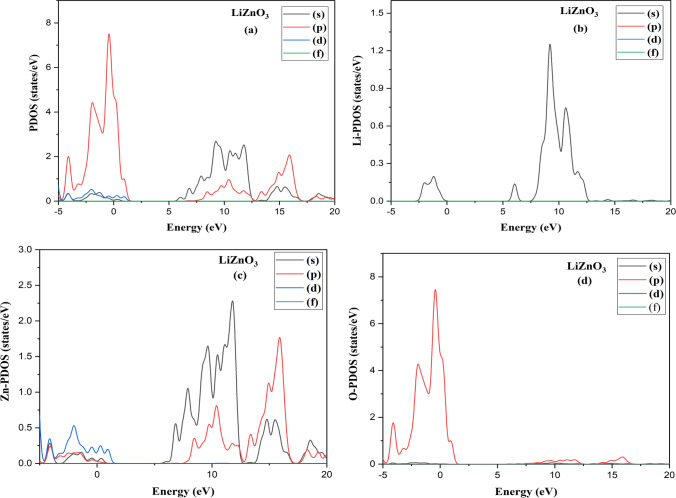
**a** Partial DOS **b** Li-PDOS and **c** Zn-PDOS, and **d** O-PDOS LiZnO_3_ compound

**Fig. 6:**
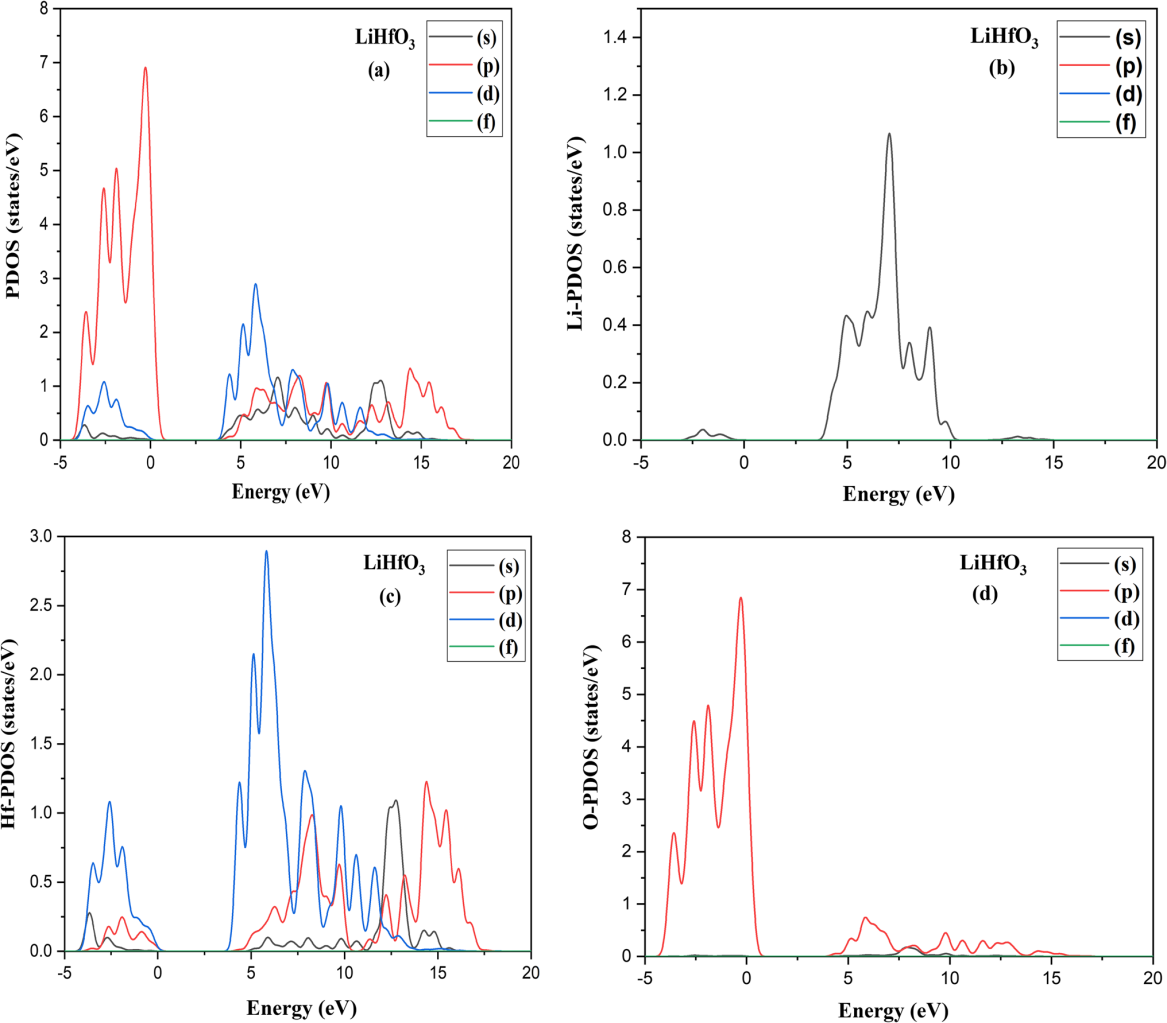
**a** Partial DOS **b** Li-PDOS and **c** Hf-PDOS, and **d** O-PDOS LiHfO_3_ compound

At 0 Kelvin temperature, it will be a semiconductor material. The plots of their DOS with respect to their band structure can be observed in Fig. 2b and 3b. The curve of LiZnO_3_ and LiHfO_3_ shows total DOS as seen in Fig. 4. The main peak of LiZnO_3_ and LiHfO_3_ compound occurs at − 0.42 eV and − 0.28 eV. The partial and elemental DOS for LiZnO_3_ and LiHfO_3_ is shown in Figs. 5 and 6. When we observe the PDOS graph, we comprehend that the main peak of p for LiHfO_3_ and LiZnO_3_ is − 0.28 eV and − 0.42 eV is the greatest peak of the compounds.

The major peak of s and d occurs at 9.19 eV and 5.82 eV and is the second greatest peak of LiZnO_3_ and LiHfO_3_. Fig. 4c and d shows the elemental PDOS for Li, Zn, Hf, and O, respectively. The oxygen (O) has a main peak at − 0.42 eV and 0.22 eV, Hf and Zn has a main peak at 5.82 eV and 11.81 eV, and Li has a main peak at 7.00 eV and 9.19 eV. The population analysis is used to better understand the bonding features of the molecule. If the bonding value is less than one, the chemical is ionic; otherwise, it is covalent. Hence, we observe the Mulliken populations values of LiHfO_3_ and LiZnO_3_ compounds in Table 2.

**Table 2:** LiHfO_3_ and LiZnO_3_ compounds Mulliken populations values

Compound Species		*s*	*p*	*d*	*f*	Total	Charge	Bond	Population	Length (Å)
LiZnO_3_	Li	2.14	0.00	0.00	0.00	2.14	0.86	O–Li	0.05	2.73
	Zn	0.30	0.87	9.76	0.00	10.93	1.07	O–Zn	0.56	1.93
	O	1.91	4.73	0.00	0.00	6.64	− 0.64	O–O	− 0.07	2.73
LiHfO_3_	Li	1.98	0.00	0.00	0.00	1.98	1.02	–	–	–
	Hf	0.33	0.48	2.11	0.00	2.93	1.07	O–Hf	1.06	2.14
	O	1.88	4.81	0.00	0.00	6.70	− 0.70	–	–	–

## Elastic constants

For the sake of applications of these compounds, various factors, such as the Birch–Murnaghan equation of state to optimize crystal structure and bond length or Goldschmidt’s rule from effective ionic radii, can determine the materials’ structural stability. The combination of anions and cations could determine the materials’ structural stability and band structure, as well as their application. The Birch–Murnaghan equation of state was applied to optimize the structural properties of the perovskites, which justified the cubic stability ground state. The elastic properties, which provide vital data about the elastic constants of solid compound, influence the crystal response to applied forces [26, 27]. The 3-independent elastic constants value is utilized to investigate the physical properties of cubic symmetry crystal, including such stability and rigidity. The values obtained of the elastic constant *C*_ij_ are observed in Table 3. From elastic property, the following relationship can be used to obtain the bulk modulus B. Mechanical stability is ensured by the elastic constants. This condition has led to the limiting condition for elastic constants to be followed; *C*_11_ > 0, *C*_44_ > 0, C_12_BC_11_, (*C*_11_–*C*_12_) > 0, (*C*_11_ + 2*C*_12_) > 0, where C_11_ is the longitudinal elastic constant that defined elasticity along unit cell’s axis and C_12_ and C_44_ are the shear elastic constants that defined elasticity in shape. The elastic constants could aid in predicting a material’s response to applied stresses [28]. Moreover, Poisson ratio (ν) is also one of the crucial parameter for providing knowledge about bonding nature. The Poisson ratio also classifies the brittle and ductile nature of solids with threshold value 0.26 that is used for separating the brittle materials from ductile ones, that is, if a material has less value than threshold, it is classified as a brittle material where more than that of threshold classify the material ductile one [29]. So, the Poisson's ratio v, Young's modulus E, and B/G (Pugh's ratio) are all presented in Table 4.

**Table 3:** Elastic constants of LiZnO_3_ and LiHfO_3_ compound

Compound	*C*_11_	*C*_12_	*C*_44_
LiZnO_3_	293.03	55.31	7.23
LiHfO_3_	156.48	42.42	15.82

**Table 4:** Compound LiZnO_3_ and LiHfO_3_ modulus

Compound	B	E	G	v	B/G
LiZnO_3_	134.55	117.07	43.20	0.35	2.11
LiHfO_3_	62.71	125.12	53.58	0.16	1.17

It is noted that a material's ductility or brittleness can be determined by using the Pugh’s ratio B/G, where B is the unit of bulk modulus and G is the unit of shear modulus. The compound is brittle if this ratio is much lower than 1.75, but ductile if it is larger than 1.75. As according to Pugh's criterion, LiZnO_3_ is ductile while LiHfO_3_ is brittle. The Poisson’s ratio (*v*) is used to calculate a compound's brittleness or ductility [30].

## Optical properties of compounds

In order to discuss the optical properties, we observed that these are strongly dependent on the electron-photon interaction occurring inside the materials. Phonons interact with electrons and exchange some energy due to their successive collisions which results in electron transport toward the conduction band. Such de-excitation involves the number of band-to-band transition which can directly affect the related optical parameters in terms of which the optical behavior of studied materials is explored. It means that optical characteristics have strong relation with electrical properties [31]. In order to further analyze the optical behavior of LiZnO_3_ and LiHfO_3_, many factor effects such as the energy loss function, ability to reflect, refractive index, absorption coefficient, and relative permittivity have been discussed in this regards.

**Fig. 7:**
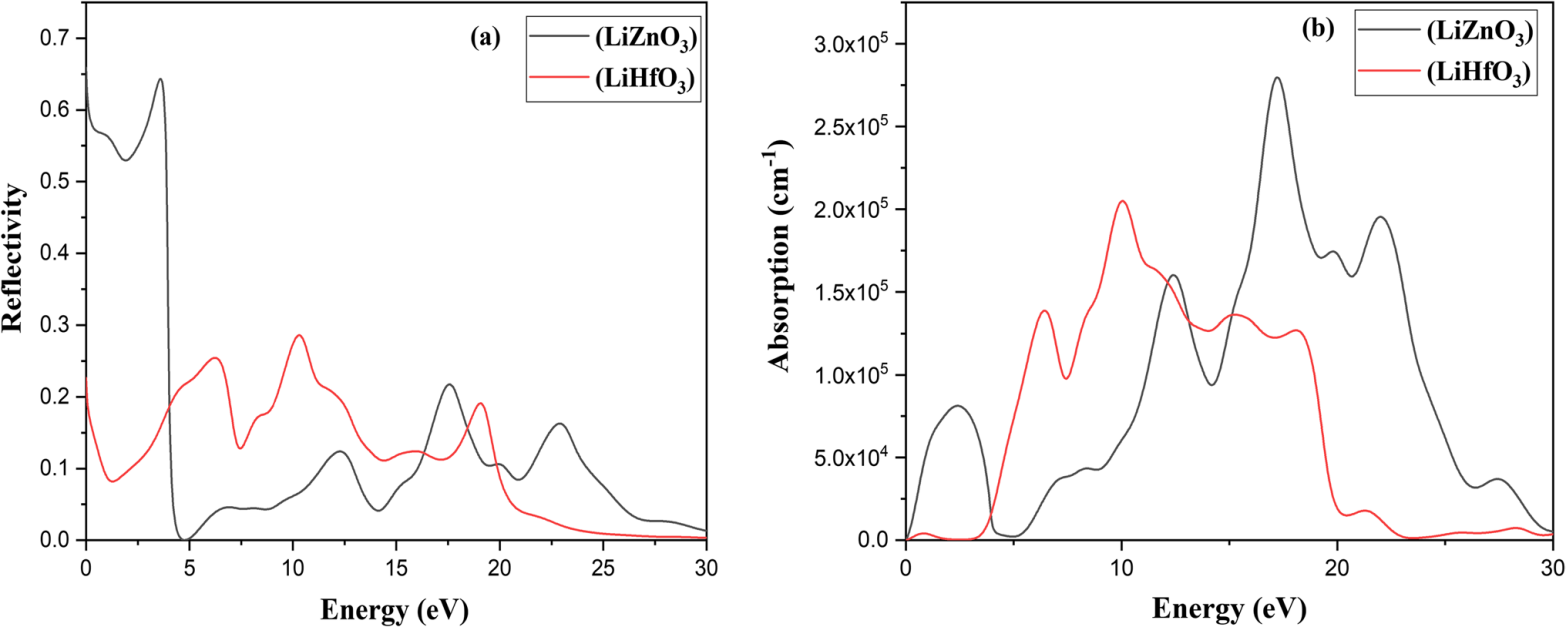
**a** Optical properties **a** Reflectivity **b** Absorption and of LiZnO_3_ and LiHfO_3_

**Fig. 8:**
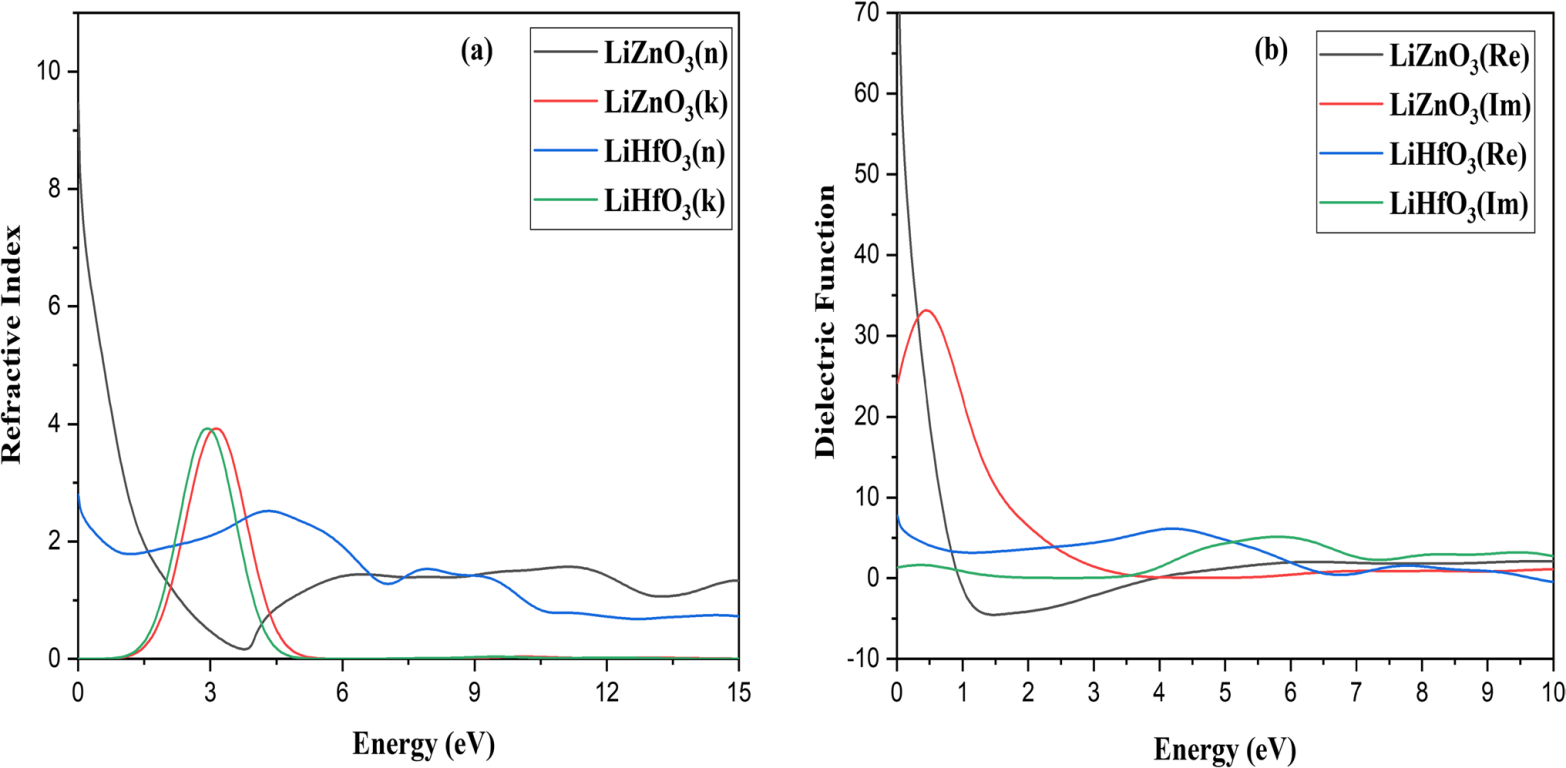
Optical properties of LiZnO_3_ and LiHfO_3_ **a** Refractive Index **b** Dielectric Function

**Fig. 9:**
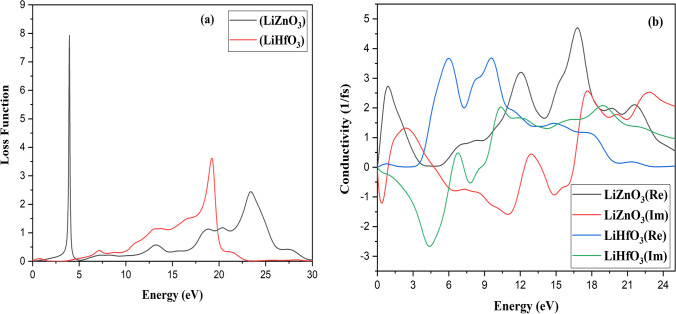
Optical properties of LiZnO_3_ and LiHfO_3_ **a** Loss Function and **b** Conductivity

These optical characteristics change with frequency. All of these features are the result of the interaction of an EM wave with a substance, which is known as wave matter interaction. The observed optical characteristics of RbZnO_3_ are seen in Figs. 7, 8, 9. The dielectric function ε(ω) is employed to study the abovementioned purpose.

To calculate the optical properties, the complex dielectric function is calculated at first using the following relation as *ε*(*ω*) = *ε*_1_(*ω*) + *iε*_2_(*ω*) [32]. The dielectric constant of a substance or material is a measure of its ability to store electrical energy. It is an expression of the extent to which a material holds or concentrates electric flux. Mathematically, dielectric constant is the ratio of a material's permittivity to the permittivity of free space. The dielectric equation's real and imaginary parts are indicated by *ε*_1_ (*ω*) and *ε*_2_ (*ω*), respectively. The real component depicts material polarization, whereas the imaginary part represents energy dissipation (loss function). Further, we observed that for the compound, *ε*_1_(*ω*) first increased with the rising value of incident electromagnetic radiation energy, which later exhibited two peaks in Fig. 8b. The first *ε*_1_ (*ω*) peak was approximately at 1.3 eV, followed by another prominent peak at 0.84 eV in the UV region. As a result, it began to decline rapidly and eventually became negative. This material exhibited metallic behavior at negative values of the real dielectric function (*ε*_1_ (*ω*)); else it is dielectric [33]. Different optical characteristics as like refractive index *n*(ω), absorption coefficient *I*(*ω*), energy loss *L*(*ω*), and reflectivity *R*(*ω*) are calculated, the related results are given in Fig. 7a for LiZnO_3_ and LiHfO_3_ compound.

The 0.66 peaks values exist at 3.55 eV and 0.28 at 10.35 eV show the major reflectivity peak for LiZnO_3_ and LiHfO_3_. At 0 eV, LiZnO_3_ and LiHfO_3_ have a reflectivity value of 0.21 and 0.65. LiZnO_3_ and LiHfO_3_ have a major absorption peak at 17.22 eV and 10.16 eV. For LiZnO_3_ and LiHfO_3_, the absorption value is 0 at 0 eV. The primary peak of LiZnO_3_ and LiHfO_3_ refractive index (*n*) is at 9.23 and 2.70 at 0.0 eV. The primary rise of LiZnO_3_ and LiHfO_3_ refractive index (*k*) is at 1.22 eV for both compounds. For LiZnO_3_ and LiHfO_3_, the primary peak of the dielectric function (*n*) emerges at 0 eV at 0 value. The dielectric function (real) for LiZnO_3_ and LiHfO_3_ is 0.33.16 at 0.43 eV and 7.81 at 0.0 eV. The complex dielectric function (imaginary) for LiZnO_3_ and LiHfO_3_ has a prominent peak around 70.0 and 7.43 at 0.0 eV. For LiZnO_3_ and LiHfO_3_, the complex dielectric function (imaginary) value at 0 eV is 0. The primary conductivity (real) spike for LiZnO_3_ and LiHfO_3_ is found at 16.67 eV and 5.96 eV. At 17.67 eV and 10.33 eV, the primary conductivity (imaginary) point for LiZnO_3_ and LiHfO_3_ occurs. LiZnO_3_ and LiHfO_3_at 0 eV have conductivity values of 0 (imaginary and real). The main max peak of loss function is 3.95 eV and 19.20 eV of LiZnO_3_ and LiHfO_3_. LiZnO_3_ and LiHfO_3_ have a loss function values of 0 at 0 eV.

## Conclusion

It is concluded that for the first time, first-principle based computations of structural adjustment, fine control of the band gap, and optical properties of oxide-perovskites, i.e., LiZnO_3_ and LiHfO_3_, have been reported. The CASTEP code, which is based on DFT and uses the GGA-PBE exchange correlation functionals and USP, was utilized to perform all calculations. The bandgap and optimized lattice parameters for LiZnO_3_ and LiHfO_3_ were found to be in good agreement. Mechanical stability criteria for the proposed materials are also met by the estimated elastic constant. Moreover, it has been observed with the aid of Pugh's criterion that LiZnO_3_ is ductile, whereas LiHfO_3_ is brittle. Furthermore, beased on the anisotropy-factor A, its onvestaged that the materials are anisotropic. It is remarkable that for LiZnO_3_ and LiHfO_3_, both VBM and CBM lay at different of each other, which results the materials in a direct band gap. In addition, the optical parameters, such as absorption, reflection, energy loss function, and refractive index, were also investigated in relation to structural elucidation. But, the imaginary element of dielectric function shows the wide energy range transparency of the proposed materials. As a result, it is possible that LiZnO_3_ and LiHfO_3_ materials could be employed in various applications such as in the photovoltaic solar cells to collect ultraviolet light, for protective rays in side windows of the cars, in the room windows and also for eye glasses.

## Data Availability

The data set used and/or analyzed in this study can be obtained from the corresponding author upon reasonable request.

## Abbreviations

DFT: Density-functional theory
CASTEP: Cambridge Serial Total Energy Package
USPPPW: Ultrasoft pseudo-potential plane-wave
BG: Indirect bandgap
PDOS: Partial density of states
TiO_2_: Titanium dioxide
UV: Ultraviolet
GPa: Giga-pascal
BZ: Brillouin zone
MPK: Monkhorst-Pack grid
MSE: Murnaghan state equation
EF: Fermi energy point
B/G: Pugh’s ratio
VB: Valance band
CB: Conduction band
VBM: Valance band maximum
CBM: Conduction band minimum
